# Supplementary material for: Trapped atoms in spatially-structured vector light fields
Source: Sci Rep. 2023 Dec 2;13:21283. doi: 10.1038/s41598-023-48589-1 (PMC10693646; doi:10.1038/s41598-023-48589-1)
Supplement: Supplementary file 1 — Supplementary Information. [file 41598_2023_48589_MOESM1_ESM.pdf]

Supplementary material

Here we provide the Mathematica code to reproduce all the calculations described in the paper. The code is organized along the following sections:

1. HG and LG beams, with various polarization structures, and propagating along the z direction are defined. Focal fields are calculated by means of equations (2),(3) and (4).

2. Longitudinal $E_z(x,y)$ and circular $E_{\sigma=\pm1}(x,y)$ electric field components of focal fields are calculated by means of equation (5). The modulus of their transverse profiles at the focal plane is shown in Fig. 1.

3. The tensor field $T^{\Delta J=2}_{\Delta m}(x,y,z)$ accounting for electronic quadrupole transition with $\Delta J = 2$, as defined in equation (12), is calculated for an arbitrary angle θ_x via equation (16). θ_x is the angle between the initial magnetic quantization axis (along z) and the rotated one. The rotation is performed around the x-axis.

4. Relative strengths are computed from equation (15) for a $\Delta J = 2$ electric quadrupole transition when the light field propagates along the magnetic quantization axis, i.e. $\theta_x = 0$. The calculations refer to the transverse section at the focal plane. The results are shown in Fig. 2.

5. Transverse cross sections of relative transitions strengths are calculated for a fixed Δm at different angles θ_x by means of equation (16). Results are shown in Fig. 3.

6. Transverse cross sections of relative strengths for motional blue sidebands of quadrupole transition are calculated via equation (20). Results are shown in Fig. 4.

1. *Light beams definition and focal fields evaluation*

```
(* Physical constants *)
Epsilon0 = 8.854187817 10^-12;      (* [J/V^2 microns] *)
c = 2.99792458 10^8;                (* [m/s] *)
HBar = 1.055 10^-34;                (* [J*s] *)
Lambda = 0.729;                     (* wavelenght [microns] *)
kz = 2Pi/Lambda;                    (* wavevector [microns^-1] *)
Omega = c kz;                       (* 2Pi frequency [Hz] *)

(* Electric field parameters *)
P0 = 10^-3;     (* power [W] *)
w0 = 1.0;       (* beam waist at the focus *)
Sigma0 = 1;     (* circular polarization *)
z0 = 10^-9;     (* numerical infinites on the exact focal plane z=0 *)

(* Optical functions *)
zR[w_] = Pi w^2/Lambda;                               (* Rayleigh length *)
R[z_,w_] = z (1 + (zR[w]/z)^2);                       (* curvature radius *)
wz[z_,w_] = w Sqrt[(1 + (z/zR[w])^2)];
El0[P_,w_] = Sqrt[4 P/(c Epsilon0 w^2)];              (* [V/m] *)
Psi[z_,w_] = ArcTan[z/zR[w]];                         (* Gouy phase *)
PsiLG[z_,p_,l_] = -(2p+Abs[l]+1)*ArcTan[z/zR[w0]];    (* Generalized Gouy phase *)

(* Electric field definitions *)
ElHG[x_,y_,z_,p_,l_] = El0[P0,w0]/Sqrt[Pi]w0/ wz[z,w0] HermiteH[p,(Sqrt[2]x)/wz[z,w0]] \
                     * HermiteH[l, (Sqrt[2]y)/wz[z,w0]] E^(-((x^2 + y^2)/wz[z, w0]^2)) \
                     * E^(I kz (x^2 + y^2)/(2 R[z, w0])) E^(I Psi[z, w0]) E^(I kz z);

ElLG[R_,Phi_,z_,p_,l_] = El0[P0,w0]/Sqrt[2]Sqrt[(2 p!)/(Pi*(p+Abs[l])!)] * w0/wz[z, w0] \
                       * ((R Sqrt[2])/wz[z, w0])^Abs[l] E^(-(R^2/wz[z,w0]^2)) \
                       * LaguerreL[p,Abs[l],(2R^2)/wz[z,w0]^2] E^(I kz R^2/(2 R[z, w0])) \
                       * E^(I l Phi) E^(I PsiLG[z, p, l]) E^(I kz z);

(* Focal fields evaluation *)
For[a = 1, a < 7, a++, {
   Which[a == 1,    (* vector beam TEM(0,0) circularly polarized Sigma=+1 *)
     u[x_,y_,z_,p_,l_] = ElHG[x, y, z, p, l];
     ElField[x_,y_,z_][a,1] = u[x,y,z,0,0];
     ElField[x_,y_,z_][a,2] = I Sigma0 u[x,y,z,0,0];
     ElField[x_,y_,z_][a,3] = I/kz (D[u[x,y,z,0,0],x] + I Sigma0 D[u[x,y,z,0,0],y]);,

     a == 2,       (* vector beam HG(0,1) circularly polarized Sigma=+1 *)
     u[x_,y_,z_,p_,l_] = ElHG[x, y, z, p, l];
     ElField[x_,y_,z_][a,1] = u[x,y,z,0,1];
     ElField[x_,y_,z_][a,2] = I Sigma0 u[x,y,z,0,1];
     ElField[x_,y_,z_][a,3] = I/kz (D[u[x,y,z,0,1],x] + I Sigma0  D[u[x,y,z,0,1],y]);,

     a == 3,       (* vector beam LG(0,1) circularly polarized Sigma=+1 *)
```

```
    u[x_,y_,z_,p_,l_] = ElLG[Sqrt[x^2  + y^2], ArcTan[x, y], z, p, l];
    ElField[x_,y_,z_][a,1] = u[x,y,z,0,1];
    ElField[x_,y_,z_][a,2] = I Sigma0 u[x,y,z,0,1];
    ElField[x_,y_,z_][a,3] = I/kz (D[u[x,y,z,0,1],x] + I Sigma0 D[u[x,y,z,0,1],y]);,

    a == 4,       (* vector beam LG(0,-1) circularly polarized Sigma=+1 *)
    u[x_,y_,z_,p_,l_] = ElLG[Sqrt[x^2  + y^2], ArcTan[x, y], z, p, l];
    ElField[x_,y_,z_][a,1] = u[x,y,z,0,-1];
    ElField[x_,y_,z_][a,2] = I Sigma0 u[x,y,z,0,-1];
    ElField[x_,y_,z_][a,3] = I/kz (D[u[x,y,z,0,-1],x] + I Sigma0 D[u[x,y,z,0,-1],y]);,

    a == 5,       (* vector beam LG(0,-1) radially polarized *)
    u[x_,y_,z_,p_,l_] = 1/Sqrt[2] ElLG[Sqrt[x^2  + y^2], ArcTan[x, y], z, p, l];
    ElField[x_,y_,z_][a,1] = u[x,y,z,0,-1] + u[x,y,z,0,1];
    ElField[x_,y_,z_][a,2] = I Sigma0 u[x,y,z,0,-1] - I Sigma0 u[x,y,z,0,1];
    ElField[x_,y_,z_][a,3] = I/kz (D[u[x,y,z,0,-1],x] + D[u[x,y,z,0,1],x] \
                           + I Sigma0 D[u[x,y,z,0,-1],y] - I Sigma0 D[u[x,y,z,0,1],y]);,

    a == 6,       (* vector beam LG(p,l) azimuthally polarized *)
    u[x_,y_,z_,p_,l_] = I/Sqrt[2] ElLG[Sqrt[x^2  + y^2], ArcTan[x, y], z, p, l];
    ElField[x_,y_,z_][a,1] = u[x,y,z,0,-1] - u[x,y,z,0,1];
    ElField[x_,y_,z_][a,2] = I Sigma0 u[x,y,z,0,-1] + I Sigma0 u[x,y,z,0,1];
    ElField[x_,y_,z_][a,3] = I/kz (D[u[x,y,z,0,-1],x] - D[u[x,y,z,0,1],x] \
                           + I Sigma0 D[u[x,y,z,0,-1],y] + I Sigma0 D[u[x,y,z,0,1],y]);]; }];
```

2. *Fig. 1: Longitudinal and circular components evaluation of focal fields*

```
For[a = 1, a < 7, a++,
SqrtInt[x_,y_,z_][a,1] = Abs[ElField[x,y,z][a,1]-I ElField[x,y,z][a,2]];
SqrtInt[x_,y_,z_][a,2] = Abs[ElField[x,y,z][a,3]];
SqrtInt[x_,y_,z_][a,3] = Abs[ElField[x,y,z][a,1]+I ElField[x,y,z][a,2]];]

scale = ConstantArray[1, {6, 3}];

For[a = 1, a < 7, a++,
 For[n = 1, n < 4, n++,
  ElMax[a, n] = FindMaximum[{SqrtInt[x, y, z0][a, n], {x, y} \[Element] Disk[{x, y}, 2 w0]}, \
                  {{x, 0.5}, {y, 0.5}}][[1]];
  If[ElMax[a, n] < 10^-5, ElMax[a, n] = 10^-10; SqrtInt[x_, y_, z_][a, n] = 0; scale[[a, n]] = 0;];\
  ]]

MaxAllEl = Max@Table[ElMax[ai, ni], {ai, 1, 6}, {ni, 1, 3}];
numPoints = 5;
Table[ContourPlot[ 1/ElMax[ai, ni] SqrtInt[x,y,z0][ai,ni], {x,-2w0,2 w0}, {y,-2w0,2w0}, ImageSize -> 270, \
      PlotPoints -> numPoints, LabelStyle -> Directive[Black, 25], PlotRange -> All, Contours -> 10, \
      ColorFunction -> ColorData[{"M10DefaultDensityGradient", {0, 1}}], ColorFunctionScaling -> False, \
      PlotLabel -> Style[Text["scale: " <> ToString[Round[MaxAllEl/ElMax[ai, ni] \
      * scale[[ai, ni]], 0.1]] <> "x"] 1,15,Black]], {ni, 1, 3}, {ai, 1, 6}]
```

3. *Tensor field evaluation*

```
WignerDl[j_,mm_,m_,Theta_] := Sum[(-1)^(k-m+mm) Sqrt[(j+m)!(j-m)!(j+mm)!(j-mm)!]/((j+m-k)!k!(j-k-mm)!(k-m+mm)!) \
                            * Cos[Theta/2]^(2j-2k+m-mm) Sin[Theta/2]^(2k-m+mm),{k,Max[0,m-mm],Min[j+m,j-mm]}]
Clear[TensorMatQ];
TensorMatQ[k_,q_,a_] = 0;
For[a = 1, a < 7, a++,

   Qm2[x_,y_,z_][a] = D[ElField[x,y,z][a,1],x] - D[ElField[x,y,z][a,2],y] \
                    + I D[ElField[x,y,z][a,2],x] + I D[ElField[x,y,z][a,1],y];

   Qm1[x_,y_,z_][a] = D[ElField[x,y,z][a,3],x] + D[ElField[x,y,z][a,1],z] \
                    + I D[ElField[x,y,z][a,3],y] + I D[ElField[x,y,z][a,2],z];

   Q0[x_,y_,z_][a] = 2/Sqrt[6] (D[ElField[x,y,z][a,1],x] + D[ElField[x,y,z][a,2],y]) \
                   + 2Sqrt[2/3] D[ElField[x,y,z][a,3],z];

   Qp1[x_,y_,z_][a]= D[ElField[x,y,z][a,3],x] + D[ElField[x,y,z][a,1],z] \
                   - I D[ElField[x,y,z][a,3],y]-I D[ElField[x,y,z][a,2], z];

   Qp2[x_,y_,z_][a] = D[ElField[x,y,z][a,1],x] - D[ElField[x,y,z][a,2],y] \
                    - I D[ElField[x,y,z][a,2],x] - I D[ElField[x,y,z][a,1],y];

   TensorMatQ[2,-2,a] = Qm2[x, y, z][a];
   TensorMatQ[2,-1,a] = Qm1[x, y, z][a];
   TensorMatQ[2,0,a] = Q0[x, y, z][a];
   TensorMatQ[2,1,a] = Qp1[x, y, z][a];
   TensorMatQ[2,2,a] = Qp2[x, y, z][a];
   ThetaTensorQ[k_,q_,a_] := Sum[WignerDl[k,qq,q,Theta] * TensorMatQ[k,qq,a],{qq,-k, k}];]
```

4. *Fig. 2: Calculation of relative strenghts for different quadrupole transitions*

```
minQ = 1/2;
mfinQ = {5/2, 3/2, 1/2, -(1/2), -(3/2)};
MaxAllEl = 0;
scale = Table[0, {ai, 1, 6}, {bi, 1, Length@mfinQ}];
For[a = 1, a < 7, a++,
 For[b = 1, b < Length@mfinQ + 1, b++,
  Clear[Theta];
  dkQ = 2;
  jinQ = 1/2;
  jfinQ = jinQ + dkQ;
  MuQ[mmQ_] = Sum[ThetaTensorQ[dkQ,q,a] ClebschGordan[{jinQ,minQ},{dkQ,q},{jfinQ,mmQ}],{q,-dkQ,dkQ}];
  Theta = N@{0};   (* [rad] *)
  MuQxy[x_,y_,z_] = MuQ[mfinQ[[b]]][[1]];
  MaxMuQ = FindMaximum[{Abs@MuQxy[x,y,z0],{x,y} \[Element]Disk[{x,y},2w0]},{{x,w0/2},{y,-(w0/2)}}][[1]];
  If[MaxMuQ > MaxAllEl, MaxAllEl = MaxMuQ];
  If[MaxMuQ > 10^-5, scale[[a, b]] = 1/ MaxMuQ, scale[[a, b]] = 0];]]

For[a = 1, a < 7, a++,
 For[b = 1, b < Length@mfinQ + 1, b++,
  Clear[Theta];
  MuQ[mmQ_] = Sum[ThetaTensorQ[dkQ,q,a] ClebschGordan[{jinQ,minQ},{dkQ,q},{jfinQ,mmQ}],{q,-dkQ,dkQ}];
  Theta = N@{0};   (* [rad] *)
  MuQxy[x_,y_,z_] = MuQ[mfinQ[[b]]][[1]];
  numPoints = 5;
  p[a,b] = ContourPlot[scale[[a,b]] Abs@MuQxy[x,y,z0],{x,-2w0,2w0},{y,-2w0,2w0}, ImageSize -> 270,\
                PlotPoints -> numPoints, LabelStyle -> Directive[Black, 15], PlotRange -> All, LabelStyle -> \
                {Plain, Black, FontSize -> 14}, Contours -> 10, ColorFunction -> \
                ColorData[{"M10DefaultDensityGradient", {0, 1}}], ColorFunctionScaling -> False, \
                PlotLabel -> Style[Text["dm=" <> ToString[mfinQ[[b]] - minQ] <> "    scale: " <> \
                ToString[Round[MaxAllEl scale[[a,b]], 0.1]] <> "x"] 1, 13, Black]]  ]]
GraphicsGrid[Table[p[ai,bi], {bi, 1, Length@mfinQ}, {ai, 1, 6}], ImageSize -> 1700]
```

5. *Fig. 3: Calculation of relative strenghts for a quadrupole transition at different interaction angles*

```
minQ = 1/2;
mfinQ = {1/2};
dkQ = 2;
jinQ = 1/2;
jfinQ = jinQ + dkQ;
ThetakB = N@{0, Pi/100, Pi/4, Pi/2};   (* [rad] *)

scale = Table[0, {ai, 1, 6}, {bi, 1, Length@ThetakB}];
For[a = 1, a < 7, a++,
 For[b = 1, b < Length@ThetakB + 1, b++,
  Clear[Theta];
  MuQ[mmQ_] = Sum[ThetaTensorQ[dkQ,q,a] ClebschGordan[{jinQ,minQ},{dkQ,q},{jfinQ,mmQ}],{q,-dkQ,dkQ}];
  Theta = ThetakB[[b]];   (* [rad] *)
  MuQxy[x_,y_,z_] = MuQ[mfinQ[[1]]];
  MaxMuQ = FindMaximum[{Abs@MuQxy[x,y,z0],{x,y} \[Element]Disk[{x,y},2w0]},{{x,w0/2},{y,-(w0/2)}}][[1]];
  If[MaxMuQ > MaxAllEl, MaxAllEl = MaxMuQ];
  If[MaxMuQ > 10^-5, scale[[a, b]] = 1/ MaxMuQ, scale[[a, b]] = 0];
  ]]

For[a = 1, a < 7, a++,
 For[b = 1, b < Length@ThetakB + 1, b++,
  Clear[Theta];
  MuQ[mmQ_] = Sum[ThetaTensorQ[dkQ,q,a] ClebschGordan[{jinQ,minQ},{dkQ,q},{jfinQ,mmQ}],{q,-dkQ,dkQ}];
  Theta = ThetakB[[b]];   (* [rad] *)
  MuQxy[x_,y_,z_] = MuQ[mfinQ[[1]]];
  numPoints = 5;
  p[a, b] = ContourPlot[ scale[[a, b]] Abs@MuQxy[x,y,z0],{x,-2w0,2w0},{y,-2w0,2w0}, ImageSize -> 270, \
                 PlotPoints -> numPoints, LabelStyle -> Directive[Black, 15], PlotRange -> All, \
                 LabelStyle -> {Plain, Black, FontSize -> 14}, Contours -> 10, ColorFunction -> \
                 ColorData[{"M10DefaultDensityGradient", {0, 1}}], ColorFunctionScaling -> False, \
                 PlotLabel -> Style[Text["dm="  <> ToString[mfinQ[[1]] - minQ] <> "    scale: " <>\
                 ToString[Round[MaxAllEl scale[[a,b]], 0.1]] <> "x"] 1, 13, Black]]; ]]
GraphicsGrid[ Table[p[ai,bi],{bi,1,Length@ThetakB},{ai,1,6}],ImageSize -> 1700]
```

6. *Fig. 4: Calculation of relative strenghts for motional sidebands of quadrupole transitions*

```
jinQ = 1/2;
minQ = 1/2;
mfinQ = 3/2;
ThetakB = N@{0};   (* [rad] *)
(* trap parameters *)
mCa = 40 1.66 10^-27;    (* 40Ca^+ approx mass [kg] *)
OmegaX = 2Pi 10^6;       (* [Hz] *)
OmegaY = 2Pi 10^6;       (* [Hz] *)
OmegaZ = 2Pi 10^6;       (* [Hz] *)
```

```
Clear[Theta]
Clear[MaxMuQ]
dkQ = 2;
jfinQ = jinQ + dkQ;
MuQ[mmQ_] = Table[Sum[ThetaTensorQ[dkQ,q,ai] \
            * ClebschGordan[{jinQ,minQ},{dkQ,q},{jfinQ,mmQ}],{q,-dkQ,dkQ}],{ai, 1, 6}];
Theta = ThetakB;   (* [rad] *)
MuQ[mfinQ][[6, 1]];
MuQxy[x_,y_,z_] = Table[MuQ[mfinQ][[ai, 1]], {ai, 1, 6}];
scale = ConstantArray[1, {6, 4}];
MaxAll = 0;
For[a = 1, a < 7, a++,
 MaxMuQ[a] = FindMaximum[{Abs@MuQxy[x,y,z0][[a]],{x, y} \[Element]Disk[{x,y},2w0]}, {{x,w0/2},{y,w0/4}}][[1]];
 If[MaxMuQ[a] > MaxAll, MaxAll = MaxMuQ[a];];
 If[MaxMuQ[a] < 10^-5, scale[[a, 1]] = 0;];
 ]

For[a = 1, a < 7, a++,
 MuQMot[x_,y_,z_][a,1] = 10^6 Sqrt[HBar/(2 mCa OmegaX)] D[MuQxy[x,y,z][[a]],x];
 MuQMot[x_,y_,z_][a,2] = 10^6 Sqrt[HBar/(2 mCa OmegaY)] D[MuQxy[x,y,z][[a]],y];
 MuQMot[x_,y_,z_][a,3] = 10^6 Sqrt[HBar/(2 mCa OmegaZ)] D[MuQxy[x,y,z][[a]],z];]

For[a = 1, a < 7, a++,
 For[n = 1, n < 4, n++,
  MaxMuQMot[a,n] = FindMaximum[{Abs@MuQMot[x,y,z0][a,n],{x,y}\[Element]Disk[{x,y},2 w0]},{{x,0.1},{y,0.2}},\
                      AccuracyGoal -> 2, PrecisionGoal -> 2][[1]];
  If[MaxMuQMot[a,n] < 10^-5, scale[[a,n+1]] = 0;]; ]]

numPoints = 5;
Table[ContourPlot[Abs[scale[[ai,1]]/MaxMuQ[ai] MuQxy[x,y,z0][[ai]]], {x,-2w0,2w0},{y,-2w0,2w0}, ImageSize -> 270, \
     PlotPoints -> numPoints, LabelStyle -> Directive[Black, 15], PlotRange -> All, Contours -> 10, ColorFunction ->\
     ColorData[{"M10DefaultDensityGradient", {0, 1}}], PlotLabel -> Style[Text["dm=" <> ToString[mfinQ - minQ] <> \
     "\!\(\*SubscriptBox[\(\[Theta]\), \(x\)]\) =" <> ToString[Round[Theta[[1]], 0.1]] <> "    scale: " <> \
     ToString[Round[MaxMuQ[1]/MaxMuQ[ai], 0.1]] <> "x"] 1, 15, Black]], {ai, 1, 6}]

Table[ContourPlot[Abs[scale[[ai,2]]/MaxMuQMot[ai, 1] MuQMot[x,y,z0][ai,1]], {x,-2w0,2w0},{y,-2w0,2w0}, \
     ImageSize -> 270, PlotPoints -> numPoints, LabelStyle -> Directive[Black, 15], PlotRange -> All, Contours -> 10,\
     ColorFunction -> ColorData[{"M10DefaultDensityGradient", {0, 1}}], PlotLabel -> Style[Text["    scale: (" <> \
     ToString[Round[(MaxMuQ[1]Sqrt[2]/w0*10^6 Sqrt[HBar/(2 mCa OmegaX)])/MaxMuQMot[ai,1],0.1]] <> \
     ")\!\(\*SuperscriptBox[\(\[Eta]x\), \(-1\)]\) x"] 1, 15, Black]],  {ai, 1, 6}]

Table[ContourPlot[Abs[scale[[ai,3]]/MaxMuQMot[ai,2] MuQMot[x,y,z0][ai,2]], {x,-2w0,2w0},{y,-2w0,2w0}, \
      ImageSize -> 270, PlotPoints -> numPoints, LabelStyle -> Directive[Black, 15], PlotRange -> All, Contours -> 10,\
      ColorFunction -> ColorData[{"M10DefaultDensityGradient", {0, 1}}], PlotLabel -> Style[Text["    scale: (" <>\
      ToString[Round[(MaxMuQ[1]Sqrt[2]/w0*10^6 Sqrt[HBar/(2 mCa OmegaY)])/MaxMuQMot[ai,2],0.1]]   <> \
      ")\!\(\*SuperscriptBox[\(\[Eta]y\), \(-1\)]\) x"] 1, 15, Black]], {ai, 1, 6}]

Table[ContourPlot[Abs[scale[[ai,4]]/MaxMuQMot[ai, 3] MuQMot[x,y,z0][ai,3]], {x,-2w0,2w0},{y,-2w0,2w0}, \
      ImageSize -> 270, PlotPoints -> numPoints, LabelStyle -> Directive[Black, 15], PlotRange -> All, Contours -> 10,\
      ColorFunction -> ColorData[{"M10DefaultDensityGradient", {0, 1}}], PlotLabel -> Style[Text["    scale: (" <>\
      ToString[Round[(MaxMuQ[1] kz*10^6 Sqrt[HBar/(2 mCa OmegaZ)])/MaxMuQMot[ai, 3], 0.1]] <>\
      ")\!\(\*SuperscriptBox[\(\[Eta]z\), \(-1\)]\) x"] 1, 15, Black]], {ai, 1, 6}]
```
